# Fluoroquinolones and glucocorticoids as risk factors for cranial cruciate ligament disease in Retrievers

**DOI:** 10.3389/fvets.2025.1625530

**Published:** 2025-07-29

**Authors:** Jessica Bunch, Kate KuKanich, Jimena Kilian, Alexa Korsberg, Butch KuKanich, Steven Martinez

**Affiliations:** ^1^College of Veterinary Medicine, Washington State University, Pullman, WA, United States; ^2^College of Veterinary Medicine, Kansas State University, Manhattan, KS, United States

**Keywords:** fluoroquinolones, corticosteroids, cruciate, ligament, Retriever

## Abstract

**Objective:**

This study aimed to determine if a higher proportion of Retrievers with cranial cruciate ligament disease (CCLD) had previous systemic exposure to fluoroquinolones or glucocorticoids compared to Retrievers without CCLD.

**Animals:**

Client-owned, Labrador and Golden Retrievers and Retriever crosses, aged 2–12 years old, were enrolled from Kansas State University and Washington State University with CCLD (cases) and without CCLD (nCCLD; healthy controls).

**Methods:**

The study was designed as a retrospective, multi-institutional case–control study. Medical records (2019–2023) were reviewed for systemic and topical exposure to fluoroquinolones or glucocorticoids within 6 months of CCLD exam or visit (nCCLD) or anytime throughout life prior to injury or visit. Data were analyzed using univariate and multivariate logistic regression analyses. The results were reported as coefficients, standard errors, *p*-values, and odds ratios with 95% confidence intervals. A *p*-value of <0.05 was considered significant.

**Results:**

A total of 419 dogs (216 cases and 203 controls) were enrolled in this study. The odds ratios of CCLD after systemic fluoroquinolone exposure were three times (CI: 1.01–8.90) higher than the odds ratios of CCLD without fluoroquinolone exposure. The odds ratios of CCLD after systemic glucocorticoid exposure were 3.51 times (CI: 1.94–6.37) higher than the odds ratios of CCLD without glucocorticoid exposure. Topical administration was not found to pose the same risk.

**Conclusion:**

The findings of this study suggest that the administration of systemic fluoroquinolones or systemic glucocorticoids was identified as a risk factor for the development of CCLD in Retriever breeds. Prospective studies are needed.

**Clinical relevance:**

Although not all risk factors for CCLD can be mitigated, systemic fluoroquinolones and/or glucocorticoids should be used cautiously in Retriever breeds.

## 1 Introduction

Cranial cruciate ligament disease (CCLD) is the most common cause of hind limb lameness in dogs (1–3). Rupture of the cranial cruciate ligament (CCL) leads to joint instability, pain, and subsequent osteoarthritis of the stifle. Loss of CCL integrity contributes to decreased canine performance, mobility, and ultimately, the quality of life. Multiple risk factors have been identified as contributing to the prevalence of CCLD in dogs, including breed, weight, conformation, early neutering, muscle imbalance, age, athletic fitness level, and other orthopedic conditions (1, 2, 4–7). To the authors’ knowledge, the evaluation of pharmaceuticals as potential clinical risk factors for CCLD in dogs has not been documented in the veterinary literature.

The use of fluoroquinolone (FQ) and glucocorticoid (GC) have been associated with tendinopathies in people (8, 9). The most commonly reported fluoroquinolone-associated tendinopathy is calcaneal (Achilles) tendon rupture, but patellar, rotator cuff, quadriceps, and hamstring tendinopathies have also been reported (8, 10, 11). In multiple studies, the odds ratio was higher for tendinopathies in individuals receiving GCs and FQs than for those receiving FQs alone (8).

*In vitro* ciprofloxacin effects on canine tendon, paratendon, and capsular fibroblast-treated cells resulted in decreased cell proliferation, collagen, and proteoglycan synthesis, as well as a significant increase in matrix-degrading proteolytic activity (12). Other *in vitro* FQ studies (involving levofloxacin and ciprofloxacin) have demonstrated cytotoxic effects on rabbit and human anterior cruciate ligament cells with enhanced apoptosis, decreased cell proliferation, and decreased extracellular matrix (13, 14). In addition to FQ-associated tendinopathies, both systemic and local GCs have been associated with delayed healing of tendons and ligaments and tendinopathies in dogs and people (9, 14–16).

The purpose of this study was to determine if exposure to FQ or GC would be associated with an increased risk of cranial cruciate ligament disease (CCLD) in Retriever and Retriever cross dogs. We hypothesized that a higher proportion of Retrievers with CCLD would have previous systemic exposure to FQ or GC compared to Retrievers without CCLD.

## 2 Methods

### 2.1 Study design

This was a retrospective multi-institutional case–control study. Medical records from Kansas State University (KSU) and Washington State University (WSU) were reviewed between 1 January 2019 and 31 December 2023 to identify cases and controls from each university’s primary care service. Information from both universities was compiled using a combination of paper and electronic medical record systems (Ezy Vet®, Instinct®, and Vetstar®).

### 2.2 Cases

Cases consisted of Labrador Retrievers, Golden Retrievers, and Retriever mixes that were presented for, or underwent, tibial plateau leveling osteotomy (TPLO) surgery at KSU or WSU between 2019 and 2023. Inclusion criteria for cases were dogs between 2 and 12 years of age at the time of CCLD diagnosis. Dogs were considered to have CCLD based on orthopedic examination by a board-certified surgeon or resident (KSU, WSU), radiographic abnormalities consistent with CCLD, and/or visualization of a damaged CCL during arthrotomy or arthroscopy. Dogs were excluded if they had evidence of hind limb orthopedic disease other than CCLD, trauma involving the hind limb, concurrent neurologic disease, concurrent hyperadrenocorticism, other concurrent acute or chronic medical diseases (e.g., other endocrinopathies, hepatic, GI, renal, and neoplasia), or if previous adequate health records could not be obtained.

### 2.3 Controls

Controls for non-CCLD diagnosed dogs (nCCLD) consisted of Labrador Retrievers, Golden Retrievers, and Retriever mixes, aged 2–12 years old, who presented to the primary care services of both universities (KSU Veterinary Health Center and WSU Community Practice) for routine care (non-orthopedic based) and had at least two visits during the study period. Dogs were excluded as nCCLD if they had evidence of hindlimb orthopedic disease (cruciate disease, any previous stifle surgery, luxating patella, trauma, osteoarthritis, and neoplasia), hyperadrenocorticism, other systemic disease, or if adequate health records could not be obtained.

### 2.4 Data collection and analysis

Adequate health records were defined as complete medication history prior to their CCLD diagnosis or primary health care visit. For both CCLD and nCCLD dogs, data regarding age, sex, neuter status, breed, weight, university enrollment center, and all prescribed systemic and topical medications were recorded, with a primary focus on FQ and GC. “Time zero” was defined as the first report of CCLD or related symptoms (e.g., limping on the hind limb, tibial thrust, cranial drawer, referral to KSU or WSU, “cruciate tear,” or “cruciate disease”) in the medical records for the CCLD dogs. For nCCLD dogs, “time zero” was defined as the last visit to the primary care service within the review period. Drug exposure within 6 months of “time zero” was defined as “6 months” for CCLD and nCCLD dogs. Patient drug exposure(s) throughout the dog’s lifetime, which also included the 6-month period before the CCLD diagnosis, were defined as “lifetime.” Following the patient’s record review, FQ and/or GC exposure was recorded as “6 months” and/or “lifetime” for both CCLD and nCCLD dogs. As drug-related orthopedic injuries most often occur in people within 1–6 months of medication administration (range 1–510 days for FQ) (8, 17, 18), this study aimed to look at both recent (within 6 months) and lifetime exposure (as defined above) to the point of CCLD diagnosis.

### 2.5 Statistical analysis

The response variable (CCLD/nCCLD) was binary. There were multiple independent variables that could be associated with the response variable, analyzed using the multivariate logistic regression analyses. Linearity of continuous variables was assessed by including the addition of the quadratic term. Multicollinearity was evaluated using the variance inflation factor, with values <2.5 being considered acceptable. Variables with a logistic univariate *p* > 0.50 were excluded from further analysis. Variables with a logistic univariate *p* < 0.50 were included in the multivariate analysis, treated as numerical or categorical as appropriate, selected according to forward selection with switching method, and retained if *p* <0.10. The results were reported as coefficient and standard error, *p*-value, and odds ratio with 95% confidence limits. Statistical significance was set at a *p* < 0.05. Calculations were performed using software Number Cruncher Statistical Systems [(v.2025), Kaysville, UT].

## 3 Results

A total of 419 dogs met the patient inclusion criteria and were enrolled into this study. There were 216 cases (CCLD) and 203 controls (nCCLD) in this study cohort.

### 3.1 Univariate analysis

The data for age, sex, breed, body weight, and enrollment center were recorded (body condition scores were not available from the data sources for this study). These independent variables underwent a univariate analysis (Table 1). KSU enrolled 240 dogs [146 CCLD (68%) and 94 nCCLD (46%)]. WSU enrollment totaled 179 dogs [70 CCLD (32%) and 109 nCCLD (54%), *p* = 0.0004].

**Table 1:** Summary [mean (SD)] and logistic univariate analysis of *P*: summary of age, body weight, reporting hospital, breed, and sex for cases and controls.

Independent variable		CCLD dogs (*N* = 216)	Non-CCLD dogs (*N* = 203)	Logistic univariate *p*-value	Variance inflation factor
Age (years)		5.82 (2.38)	5.97 (2.84)^*^	0.001	1.10
Body weight (kg)		37 (9)^*^	32 (8)	<0.0001	1.22
Veterinary teaching hospital study sites	KSU^*^	146 (68%)	94 (46%)	0.0004	1.10
	WSU	70 (32%)	109 (54%)		
Breed	Labrador Retriever^*^	117 (54%)	80 (39%)	0.007	1.84
	Golden Retriever	36 (17%)	50 (25%)	0.51	1.84
	Retriever Cross	63 (29%)	73 (36%)		
Sex	Male total	88 (41%)	98 (45%)	0.12 (male vs. female, intact vs. neutered/spayed)	1.13 (female vs. male) 1.10 (intact vs. neutered/spayed)
	Male intact	6 (2.8%)	15 (7.4%)		
	Male neutered	82 (38%)	83 (41%)		
	Female total	128 (59%)	105 (49%)		
	Female intact	4 (1.8%)	2 (1.0)		
	Female spayed	124 (57%)	103 (51)		

CCLD, cranial cruciate ligament disease; SD, standard deviation; yrs, years; kg, kilogram; KSU, Kansas State University; WSU, Washington State University. ^*^Statistical significance.

There were multiple independent variables identified with the univariate analysis to have significance in relation to the occurrence of CCLD. Dogs in this study with CCLD were significantly younger (mean = 5.82 years) than nCCLD dogs (mean = 5.97 years), *p* = 0.001. CCLD dogs also had a significantly higher mean body weight (37 kg) than nCCLD dogs (32 kg; *p* < 0.0001). A higher proportion of CCLD dogs were Labrador Retrievers (117/218, 54%), *p* = 0.007, compared to Golden Retrievers (36/218, 17%) and Retriever crosses (63/218, 29%). Although the population of female dogs to male dogs with CCLD was greater in this study (*F* = 128/216, 59%; M = 88/216, 41%), no significant differences were noted for intact/neuter status in relation to CCLD, *p* = 0.12.

The univariate analysis was also performed for all the GC, FQ, and combination of GC and FQ routes and their exposure times in both patient groups (Table 2). There were no significant effects noted for CCLD development in dogs with systemic or topical FQ, GC, or FQ + GC drug exposures within 6 months of CCLD diagnosis. However, significant effects were identified between drug exposures occurring anytime during the dog’s lifetime prior to CCLD diagnosis or control visit for systemic FQ (*p* = 0.02), systemic GC (*p* = 0.0001), and systemic FQ + GC (*p* = 0.03). There was no association with topical drug exposure anytime during life and the development of cruciate disease.

**Table 2:** Summary and logistic univariate analysis of FQ and GC route and timing of administration by case (CCLD) and control (non-CCLD).

Administration route in relation to “time zero”^a^	CCLD cases and %	Non-CCLD cases and %	Logistic univariate *p*-value	Variance inflation factor
Systemic FQ within 6 months	2 (0.9)	1 (0.5)	0.61	1.42
Systemic FQ lifetime^*^	18 (8.3)	6 (3.0)	0.02	2.20
Topical FQ within 6 months	4 (1.8)	4 (2.0)	0.93	1.34
Topical FQ lifetime	26 (11.9)	25 (12.3)	0.82	9.38
Systemic GC within 6 months	10 (4.6)	3 (1.5)	0.08	1.16
Systemic GC lifetime^*^	73 (33.5)	25 (12.3)	<0.0001	1.40
Topical GC within 6 months	33 (15.1)	29 (14.3)	0.78	1.35
Topical GC lifetime	117 (53.7)	93 (45.8)	0.11	1.23
FQ + GC systemically within 6 months	0 (0)	0 (0)	–	–
FQ + GC systemically in lifetime^*^	11 (5.1)	2 (1.0)	0.03	2.38
FQ + GC topically within 6 months	4 (1.8)	4 (2.0)	−0.93	−0.00
FQ + GC topically in lifetime	24 (11.0)	25 (12.3)	−0.49	−1.21

CCLD, cranial cruciate ligament disease; FQ, fluoroquinolone; GC, glucocorticoid; OR, odds ratio. ^*^Indicates significance.

a “Time zero”, defined as the first mention of CCLD or related symptoms in the medical records for cases. For controls, “time zero” was the last visit to the primary care service within the review period.

Multicollinearity was identified with several independent variables for FQ, GC, and FQ + GC exposures at 6 months and lifetime, resulting in variance inflation factors (VIFs) > 2.5. To produce a final multivariate analysis with only non-multicollinearity variables, those variables with VIFs > 2.5 or *a p*-value > 0.50 were excluded from the multivariate analysis. The multicollinearity correction also added the following independent variables remaining for multivariate analysis: systemic GC exposure within 6 months, topical GC for lifetime, and topical FQ + GC for lifetime exposure in CCLD diagnosis (Table 3).

**Table 3:** Corrected logistic univariate analysis for non-multicollinearity independent variables^a^ (following variable exclusion of VIF > 2.5 and *p* > 0.50).

Independent variable	Variance inflation factor
Hospital Study Center (KSU vs. WSU)	1.09
Age (years)	1.09
Sex: female vs. male	1.13
Sex: intact vs. neutered/spayed	1.09
Weight (kg)	1.21
Systemic FQ for lifetime	2.20
Systemic GC for 6 months	1.16
Systemic GC for lifetime	1.40
Topical GC for lifetime	1.23
Systemic FQ + GC for lifetime	2.38
Topical FQ + GC for lifetime	1.21
Labrador Retrievers	1.82
Other breeds (Golden Retrievers, Retriever Cross)	1.83

VIF, variance inflation factor; FQ, fluoroquinolone; GC, glucocorticoid.

a following independent variable exclusion of VIF > 2.5 and *p* > 0.50.

### 3.2 Multivariate analysis

The remaining independent variables (non-multicollinearity, VIF < 2.5, logistic univariate *p* < 0.5) were used to perform the multivariate logistic regression analysis on CCLD and nCCLD patient populations using forward selection with switching, with independent variables retained if *p* < 0.10. The analysis included controlling for the independent factors of veterinary teaching hospital site, patient age, weight, sex, and breed (Table 4).

**Table 4:** Multivariate logistic regression of non-multicollinearity independent variables (VIF < 2.5, *p* < 0.10; reported with the results of only significant independent variables).

Independent variable	Independent variable	Standard error	*p*-value	Odds ratio	95% Confidence interval limits for odds ratio	
					Lower	Upper
Hospital study center (KSU vs. WSU)	0.824	0.233	0.0004	2.280	1.444	3.599
Age (years)	0.593	0.233	0.011	1.809	1.147	2.854
Sex: female vs. male	0.750	0.240	0.002	2.116	1.322	3.386
Weight (kg)	0.080	0.016	0.0001	1.083	1.050	1.118
Systemic GC for lifetime	1.256	0.303	0.0001	3.513	1.938	6.367
Systemic FQ for lifetime	1.100	0.554	0.047	3.005	1.014	8.904
Labrador Retrievers	0.653	0.309	0.035	1.921	1.049	3.521

FQ, fluoroquinolone; GC, glucocorticoid.

**Figure 1:**
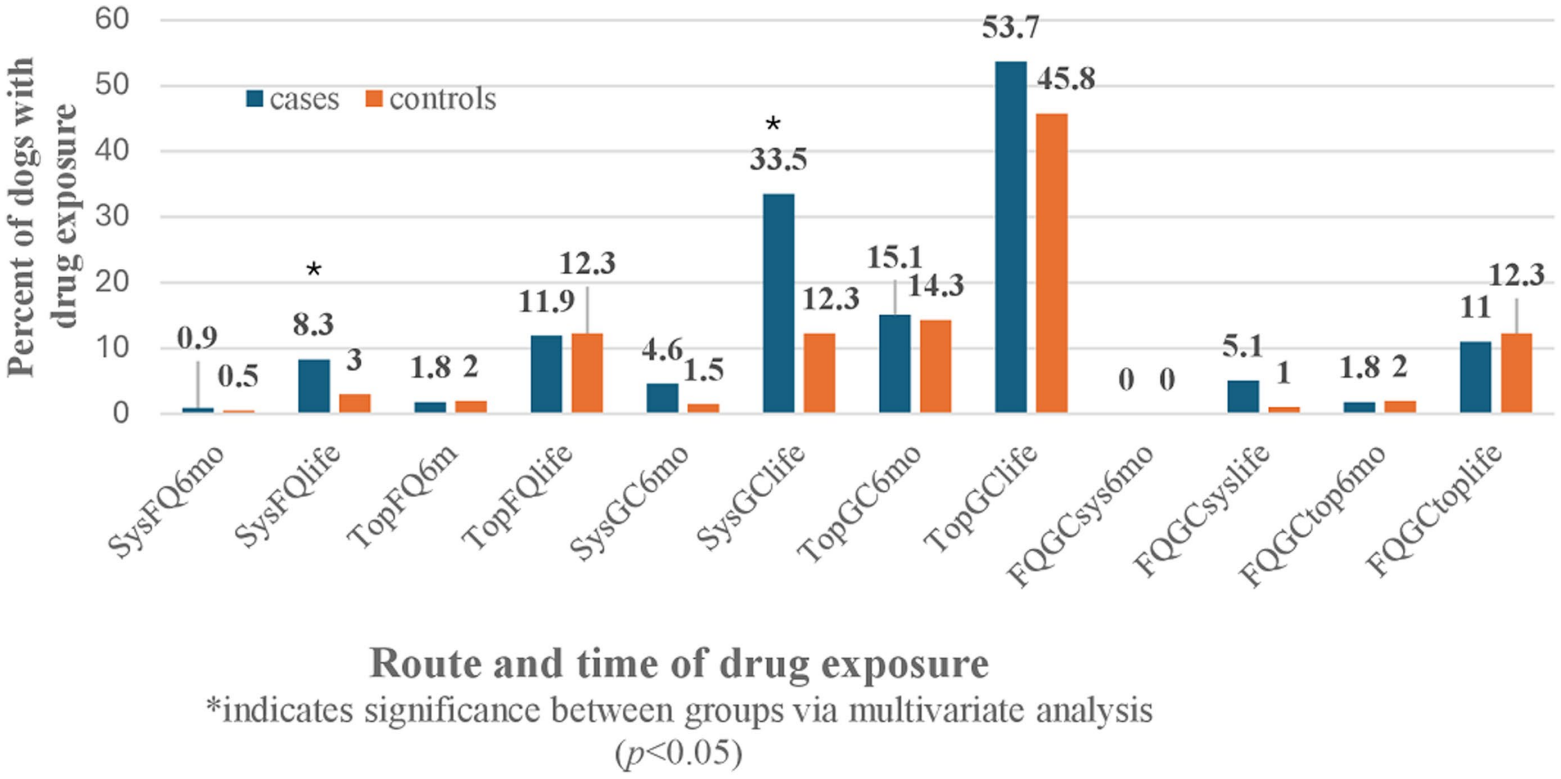
Drug exposure time and route.

The odds ratio of developing CCLD were 3.5 times greater after systemic GC exposure at any point during a Retriever’s lifetime prior to injury compared to no exposure to the drug (OR: 3.513, CI: 1.938–6.367, *p* = 0.0001). The odds ratio of developing CCLD were three times greater after systemic FQ exposure at any point during a Retriever’s lifetime prior to injury compared to no exposure to the drug (OR: 3.005, CI: 1.014–8.904, *p* = 0.047). No statistically significant effects were identified from exposure to topical or systemic FQ, GC, or FQ + GC formulations during the 6 months preceding the diagnosis of CCLD in Retrievers. There were also no effects identified from lifetime exposure to topical FQ or GC among Retrievers during CCLD development (Figure 1).

## 4 Discussion

To the authors’ knowledge, this is the first study examining exposure to FQ or GC as a possible risk factor for the development of CCLD in Retrievers. The results support our hypothesis that a higher proportion of Retrievers with CCLD would have previous exposure to systemic FQ or GC than Retrievers without CCLD.

Fluoroquinolone-associated tendinopathies in humans can occur in patients aged between 18 and 91 years but are more common in older patients with a mean age of 59 years (18). Other risk factors include renal dysfunction, solid organ transplant, and concurrent use of low-dose corticosteroids, with reports of up to 52.5% of affected patients receiving corticosteroids (18–20). Although the prevalence of FQ-associated tendinopathies is low (0.14–0.4%), the use of FQ carries a 3.8-fold increased risk compared to other antibiotics, and due to the severity of the condition, FQ is accompanied by a “black box” product warning label for tendonitis and tendon rupture (18–20). The current guidelines for athletes are to avoid FQ use unless no alternative is available, and systemic corticosteroids should not be used concomitantly (18). The median duration of fluoroquinolone use prior to the development of tendinopathies in humans is 8 days, but symptoms have been reported as quickly as 2 h after the first dose or as long as 6 months after administration, with a reported range of 1–510 days for FQ (18–21). Based on these data, we designed our study to evaluate both the time period of 6 months prior to “time zero” and lifetime exposure. Although we did not find an increased risk with those patients receiving FQ within 6 months of CCLD presentation, this may have been due to the smaller number of patients receiving systemic FQ in our canine patient population. Additionally, no patients in our study received both systemic FQ and GC within 6 months of the reported injury. Without further research and more cases with this timeframe of exposure, it is difficult to ascertain if more recent exposure increases the risk of a drug-associated tendinopathy and/or ligament damage in the canine patient. Other factors that need to be considered include the differences in absorption, distribution, metabolism, and excretion of these drugs in canines compared to humans. The fact that CCLD is a degenerative disease in dogs could also explain why lifetime exposure to FQ or GC would have an effect in canines but not necessarily in humans (e.g., lifetime risks may be cumulative in dogs).

The use of GCs significantly increases the risk of FQ-associated tendinopathies in people and is believed to synergistically potentiate the development of the FQ-related tendinopathy (18). When used concurrently with FQs, GCs have been associated with a 46-fold greater risk of calcaneal (Achilles) tendon rupture compared to individuals taking neither medication (18). GCs have been associated with different types of tendon disorders; however, 41% of reported cases in humans had underlying conditions in which tendon rupture may be a feature of the disease (9). Given the prevalence of GC use in veterinary medicine and their close association with FQ-associated tendinopathies in people, we investigated whether this could also prove a risk factor for CCL injuries. The effects of long-term and high-dose administration of GC on the CCL in healthy Beagles demonstrated that GC may influence metabolism (mucopolysaccharide and elastic fiber production), and these changes had a small effect on the strength of the otherwise healthy ligament (16). These changes were different from the degeneration observed in spontaneous CCLD; therefore, it was unclear from this study if prolonged exposure to GC is a risk factor for CCLD (16). Given our findings, GC may play a role in CCLD when administered systemically in dogs at risk for CCLD and, therefore, should be investigated further, particularly if the ligament has other underlying pathologies. Although we had more Retrievers with exposure to GC within 6 months of CCLD diagnosis than those with exposure to FQ, it did not reach significance. This finding could indicate that the time from exposure is less of an issue compared to the frequency of exposure and/or dose. Further studies should be conducted to further elucidate these possibilities.

Tendons and ligaments are closely related fibrous connective tissues that anatomically differ in location and function. Both tissue types are composed primarily of water and an extracellular matrix (ECM) primarily made of type 1 collagen fibers (22, 23). There are some differences in ECM composition and organization varying by individual tendon, ligament, and anatomical location, but structurally and physiologically, they are very similar. It is interesting to note that the use of FQs and GCs may have very different effects on collagen between species. In people, the effects of FQ and GC exposure appear to be more deleterious to tendons, with fewer case studies reported for FQ-associated ligament injuries (10), although *in vitro* studies have shown FQs and GCs to have a negative metabolic effect on human ligament cells, inducing cell apoptosis and cellular senescence (14). Although *in vitro* examination of dog tendons shows that FQs can result in degenerative pathology (12), the clinical effect of FQs and GCs from the results of this study suggests that there may be a greater effect for the potential of ligament disease in the development of CCLD.

Studies and case reports investigating the effects of FQs and GCs on tendons and ligaments are predominantly focused on the systemic use of these medications. Although sporadic reports of topical or local application of GCs may contribute to tendinopathies in people, the majority of literature supports that those routes of administration have a much lower risk in the development of tendon or ligament injuries (9). Topical drug administration commonly results in high concentrations at the site of administration but much lower systemic drug concentrations, thereby decreasing the risk for systemic adverse effects. Our findings support this finding, as we did not find a significantly increased risk of CCLD with the topical administration of FQ or GC.

Limitations of this study include its retrospective nature and dependence on the quality and detail of medical records, but this was not expected to influence data analysis, as patients with incomplete records were excluded from the study. Although nCCLD-diagnosed dogs were evaluated by primary care faculty veterinarians in a university setting, it was not a requirement for these patients to have evaluations performed by a board-certified small animal surgeon, a canine sports medicine and rehabilitation specialist, or residents, and therefore, some cases with mild CCLD could have been missed. We believe the risk of this effect is low given that nCCLD patients were not presenting for lameness, were undergoing a full physical examination by skilled faculty veterinarians, and consultations with boarded surgeons or residents would have been available onsite if there were concerns of CCLD. CCLD and nCCLD dogs were not age, sex, or weight matched as we did not have an even number of patients in these groups. We also limited the inclusion criteria to Retrievers due to the known increased incidence of CCLD in Labrador Retrievers, but included Golden Retrievers and Retriever mixes, as this was not a prevalence study but rather an investigation into risk factors. Breeds were self-reported as such by owners; therefore, breed accuracy could not be confirmed. CCLD is not limited to the Retriever breeds; however, even with these initial findings, we cannot necessarily apply our findings to other breeds without further research.

Significantly more CCLD dogs were enrolled from KSU compared to WSU. Since the CCLD population in this study consisted of cases that were referred to each veterinary teaching hospital for a TPLO surgery, it is likely that there are several factors affecting this finding. These factors could include owner demographics, surrounding population, breed prevalence, hospital variables, and the prevalence of surrounding surgical facilities. Additionally, our enrollment period did coincide with the COVID-19 pandemic, which could have affected each hospital’s operations differently.

For this study, specific drugs (generic or brand names) within each category of FQ or GC were not individually assessed for risk. Therefore, we were not able to determine if a specific drug, or drugs within the drug class, produced a greater risk. Drug dose, frequency, and length of use were also not assessed and beyond the scope of this study, as this was an initial investigation of risk factors from which future prospective studies could be designed. Future prospective studies could assess individual drugs within each class and the potential role of dose, frequency, and length of use for the risk of CCLD. Additionally, these studies could control for concurrent medications or further investigate any potential negative or positive synergistic effects of concurrent drug use at varying dosages.

Body condition score was not included as a variable since it was not consistently reported; however, dogs that were heavier were at an increased risk of CCLD, and Labrador Retrievers were at higher risk. These findings are consistent with results from previously published studies (1, 4–6, 24–26). To ensure these known risk factors and other independent variables did not affect our findings, a multivariable logistic regression was performed, and multicollinearity was assessed. Studies have also demonstrated that age at neutering is another factor to consider when assessing risk for CCLD (4, 7). This was not addressed in our study, and we did not record this data in our analysis, which is a limitation and should be included in future studies.

Recommendations for future studies would include a larger sample size, matching for age, sex, weight, assessing individual or combination drugs, topical or systemic formulations, dosages, and duration within each drug class, capturing body condition score and age at neutering, as well as investigating other breeds. Prospective studies would be ideal, as they could allow for control of co-morbidities, which was not performed in this study beyond ensuring no known cases of hyperadrenocorticism could be identified from the medical record, which would be a concern as a confounder given the increased concentration of endogenous glucocorticoids with this condition. It should also be noted that some of the CCLD and nCCLD patients in this study were on multiple systemic medications, and the role that these drugs may have had in the overall effect on the CCLD patient population selected is unknown. Therefore, controlling for other drugs administered would be beneficial in future studies.

CCLD development was chosen as the primary condition to investigate any potential risks from exposure to FQs and GCs. Although ligaments and tendons are histologically and biochemically very similar to each other, the medical literature mainly cites the negative effects of FQ and GC on tendons, with the sequelae of tendinopathies. Without further veterinary research, it would be a non-supported statement to suggest that FQ and GC may negatively affect ligaments over tendons in dogs. Therefore, future studies should also evaluate the effects of FQ and GC on calcanean and other tendinopathies in dogs.

In conclusion, lifetime exposure to systemic FQ or GC may be an additional risk factor for developing CCLD in Retrievers. Although systemic administration of these medications may be unavoidable in some cases, alternative medications should be considered, especially for canine athletes, hunting, or working Retriever. Additionally, given the importance of antimicrobial stewardship, FQ should not be considered a first-line antibiotic.

## Data Availability

The original contributions presented in the study are included in the article/supplementary material. Further inquiries can be directed to the corresponding author.
